# Improving squalene production by enhancing the NADPH/NADP^+^ ratio, modifying the isoprenoid-feeding module and blocking the menaquinone pathway in *Escherichia coli*

**DOI:** 10.1186/s13068-019-1415-x

**Published:** 2019-03-28

**Authors:** Wen Xu, Jia Yao, Lijun Liu, Xi Ma, Wei Li, Xiaojing Sun, Yang Wang

**Affiliations:** The Molecular Virology and Viral Immunology Laboratory, Xi’an Medical University, Xi’an, 710021 Shaanxi China

**Keywords:** Squalene, UdhA, *pgi*, Feeding module modification, Rate-limiting enzymes, Menaquinone pathway

## Abstract

**Background:**

Squalene is currently used widely in the food, cosmetics, and medicine industries. It could also replace petroleum as a raw material for fuels. Microbial fermentation processes for squalene production have been emerging over recent years. In this study, to study the squalene-producing potential of *Escherichia coli* (*E. coli)*, we employed several increasing strategies for systematic metabolic engineering. These include the expression of human truncated squalene synthase, the overexpression of rate-limiting enzymes in isoprenoid pathway, the modification of isoprenoid-feeding module and the blocking of menaquinone pathway.

**Results:**

Herein, human truncated squalene synthase was engineered in *Escherichia coli* to create a squalene-producing bacterial strain. To increase squalene yield, we employed several metabolic engineering strategies. A fivefold squalene titer increase was achieved by expressing rate-limiting enzymes (IDI, DXS, and FPS) involved in the isoprenoid pathway. Pyridine nucleotide transhydrogenase (UdhA) was then expressed to improve the cellular NADPH/NADP^+^ ratio, resulting in a 59% increase in squalene titer. The Embden–Meyerhof pathway (EMP) was replaced with the Entner–Doudoroff pathway (EDP) and pentose phosphate pathway (PPP) to feed the isoprenoid pathway, along with the overexpression of *zwf* and *pgl* genes which encode rate-limiting enzymes in the EDP and PPP, leading to a 104% squalene content increase. Based on the blocking of menaquinone pathway, a further 17.7% increase in squalene content was achieved. Squalene content reached a final 28.5 mg/g DCW and 52.1 mg/L.

**Conclusions:**

This study provided novel strategies for improving squalene yield and demonstrated the potential of producing squalene by *E. coli*.

**Electronic supplementary material:**

The online version of this article (10.1186/s13068-019-1415-x) contains supplementary material, which is available to authorized users.

## Background

Squalene intake becomes effective through the everyday diet or after intravenous injection, which appears to be critical in reducing incidence of coronary heart disease and cancers [1, 2]. If it could be produced sustainably on a large scale, it could also be used instead of petroleum as a raw material for fuels [3, 4]. Therefore, research into producing squalene via microbial fermentation has gained significant interest over recent years. Natural squalene producers include *Saccharomyces cerevisiae, Aurantiochytrium mangrovei, Schizochytrium mangrovei, Thraustochytrid*, cyanobacterium *Synechocystis* and *Rhodopseudomonas palustris,* which have been extensively studied for their squalene production [5–15]. However, squalene yields from these microorganisms are still low compared to *Amaranthus* seed oil and olive oil [16]. Novel microorganisms and development strategies are required for higher microbial squalene yields. *Aurantiochytrium* sp. 18W-13a and Yonez 5-1 can accumulate up to 20% and 32% DCW, respectively, as squalene [17, 18]. However, genetic engineering of these organisms remains a daunting challenge. Therefore, it is difficult to modify the triterpenoid pathways in these organisms to obtain greater squalene yields than currently possible. Alternatively, *Saccharomyces cerevisiae* and *Escherichia coli* can be engineered for squalene overproduction due to their favorable fermentation characteristics and well-understood genetic editing tools.

**Fig. 1:**
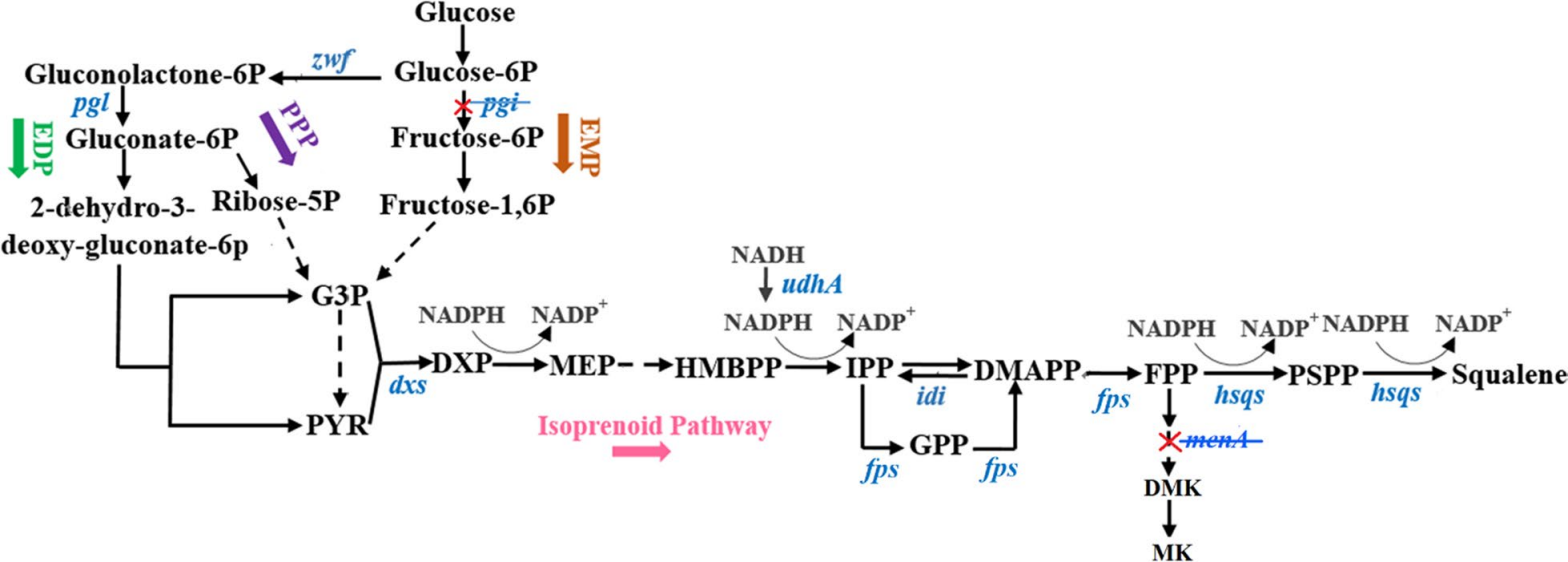
Biosynthesis of squalene through isoprenoid pathway and the feeding modules. *DMAPP* dimethylallyl diphosphate, *dxs* encoding DXP synthase, *DXP* 1-deoxy-d-xylulose-5-phosphate, *EDP* Embden–Meyerhof pathway, *EMP* Entner–Doudoroff pathway, *FPP* farnesyl diphosphate, *GPP* geranyl diphosphate, *G3P* d-glyceraldehyde-3-phosphate, *HMBPP* (E)-4-hydroxy-3-methyl-but-2-enyl pyrophosphate, *hsqs* encoding squalene synthease, *idi* encoding IPP isomerase, *IPP* isopentenyl diphosphate, *fps* farnesyl diphosphate synthase, *MEP* methylerythritol 4-phosphate, *pgi* encoding glucose-6-phosphate isomerase, *pgl* encoding 6-phosphogluconolactonase, *PPP* pentose phosphate pathway, *PSPP* pre-squalene diphosphate, *PYR* Pyruvate, *udhA* encoding the soluble pyridine nucleotide transhydrogenase, *zwf* glucose-6-phosphate dehydrogenase, dashed arrow indicates multiple enzymatic steps

In the previous study, *E. coli* JM109 (DE3) was used for heterologous squalene production via expression of human truncated squalene synthase [19]. Squalene synthase catalyzes the fusion of two farnesyl diphosphate (FPP) molecules from the isoprenoid pathway into one squalene molecule using two consecutive steps [20]. To improve product titer, a common strategy is to elevate the expression level of rate-limiting enzymes to enhance pathway flux. Herein, the rate-limiting enzymes involved in the isoprenoid pathway 1-deoxyxylulose-5-phosphate synthase (DXS), farnesyl diphosphate synthase (FPS) and isopentenyl diphosphate isomerase (IDI) [21–24], were overexpressed to direct flux towards FPP (Fig. 1).

Since many industrially useful compounds require NADPH for synthesis, its availability is essential for metabolic engineering [25–30]. Several metabolic engineering strategies for cofactors NADH and NADPH have recently been reported [31–35]. 1-Deoxy-d-xylulose-5-phosphate reductoisomerase (DXR), 4-hydroxy-3-methylbut-2-enyl diphosphate reductase (IspH) and squalene synthase (SQS) have been reported to require NADPH as a cofactor (Fig. 1), therefore the ratio of the cofactor pair NADPH/NADP^+^ appears important in regulating squalene synthesis [36, 37]. However, the effect of NADPH availability on squalene synthesis has yet to be reported. Herein, we expressed a soluble pyridine nucleotide transhydrogenase (UdhA) that catalyzes reversible hydride transfer between NAD(H) and NADP(H) to maintain homeostasis of these redox cofactors [26]. This was to increase the ratio of NADPH/NADP^+^ to favor the biosynthesis of squalene.

To improve the FPP pool for squalene synthesis, flux to the isoprenoid pathway should be enhanced. The isoprenoid pathway, fed by the Embden–Meyerhof pathway (EMP), starts with the condensation of pyruvate and glyceraldehyde-3-phosphate (G3P) to form 1-deoxy-d-xylulose-5-phosphate (DXP) (Fig. 1). However, the isoprenoid pathway is limited by EMP due to imbalanced generation of pyruvate and G3P precursors [38]. It has been proved that the Entner–Doudoroff pathway (EDP) combined with the pentose phosphate pathway (PPP) was an ideal feeding module for the isoprenoid pathway [39]. Therefore, in this study, the *pgi* gene was deleted to block the EMP and activate the EDP and PPP [40] to supply more precursors for the isoprenoid pathway. Notably, rate-limiting enzymes in the EDP and PPP, such as glucose-6-phosphate dehydrogenase and 6-phosphogluconolactonase, are regulated by oxidative inducers to maintain low activities [41]. Thus, *zwf* and *pgl* genes encoding the two enzymes were expressed to minimize the bottleneck from the EDP and PPP (Fig. 1).

Menaquinone (MK) and demethylmenaquinone (DMK) function as electron transporters in the anaerobic respiratory chain in *E. coli* [42]. Although the MK and DMK have no essential function for aerobiosis of *E. coli*, they are synthesized at a high concentration under aerobic condition [43]. As shown in Fig. 1, DMK/MK synthesis occurs as a branched pathway of squalene synthesis in *E. coli*, suggesting that MK pathway and squalene pathway inhibit each other by competing for the common precursor FPP. Thus, theoretically, blocking the MK pathway by deleting *menA* gene may facilitate squalene accumulation by decreasing the consumption of FPP.

In this study, truncated human squalene synthase was introduced to *E. coli* JM109 (DE3) to produce squalene. Rate-limiting enzyme overexpression, as a classic engineering strategy, was used to direct carbon flux towards squalene biosynthesis. The ratio of NADPH/NADP^+^ was enhanced by expressing UdhA to improve the activities of enzymes involved in squalene synthesis. Moreover, the feeding module for the isoprenoid pathway was modified by blocking EMP and activating EDP and PPP pathways, accompanied by the overexpression of rate-limiting enzymes. Mk pathway was blocked by *menA* gene deletion to save more FPP for squalene synthesis. Results indicate that the strategies presented here are efficient for increasing squalene production.

## Materials

### Strains, reagents, and shake flask cultivation

*Escherichia coli* JM109 (DE3) (Promega, USA) was used as the host for metabolic engineering. The standards of squalene and MK were purchased from Sigma-Aldrich (Shanghai, China). Restriction enzymes, Taq polymerase and T4 ligase were purchased from Takara (Dalian City, China). Gel extraction kit, PCR purification kit and plasmid purification kit were purchased from QIAGEN (Hilden, Germany). A high-performance liquid chromatography system (Hitachi, Tokyo, Japan) was used for squalene analysis. Engineered strains for squalene overproduction were cultured at 37 °C in LB medium (10 g/L NaCl, 10 g/L peptone, 5 g/L yeast extract) supplemented with 10 g/L glucose and 1.0 g/L MgSO_4_·7H_2_O. 100 mg/L ampicillin and 50 mg/L streptomycin were added to culture medium to retain plasmids with corresponding antibiotic selection markers. For squalene production, all *E. coli* strains were cultured in 250 mL shake flasks supplemented with 50 mL culture media on a rotary shaker at 200 rpm. After 2 h of incubation, when the cell cultures reached an OD_600_ of 0.5–0.6, 0.5 mM isopropyl-1-thio-β-d-galactopyranoside (IPTG) was added to induce expression of genes under the control of IPTG-inducible promoters. Cell cultures were grown for a further 32 h before harvesting and analyzing for squalene content. All strains used in the present study are listed in Additional file 1: Table S1.

### Plasmid construction

**Fig. 2:**
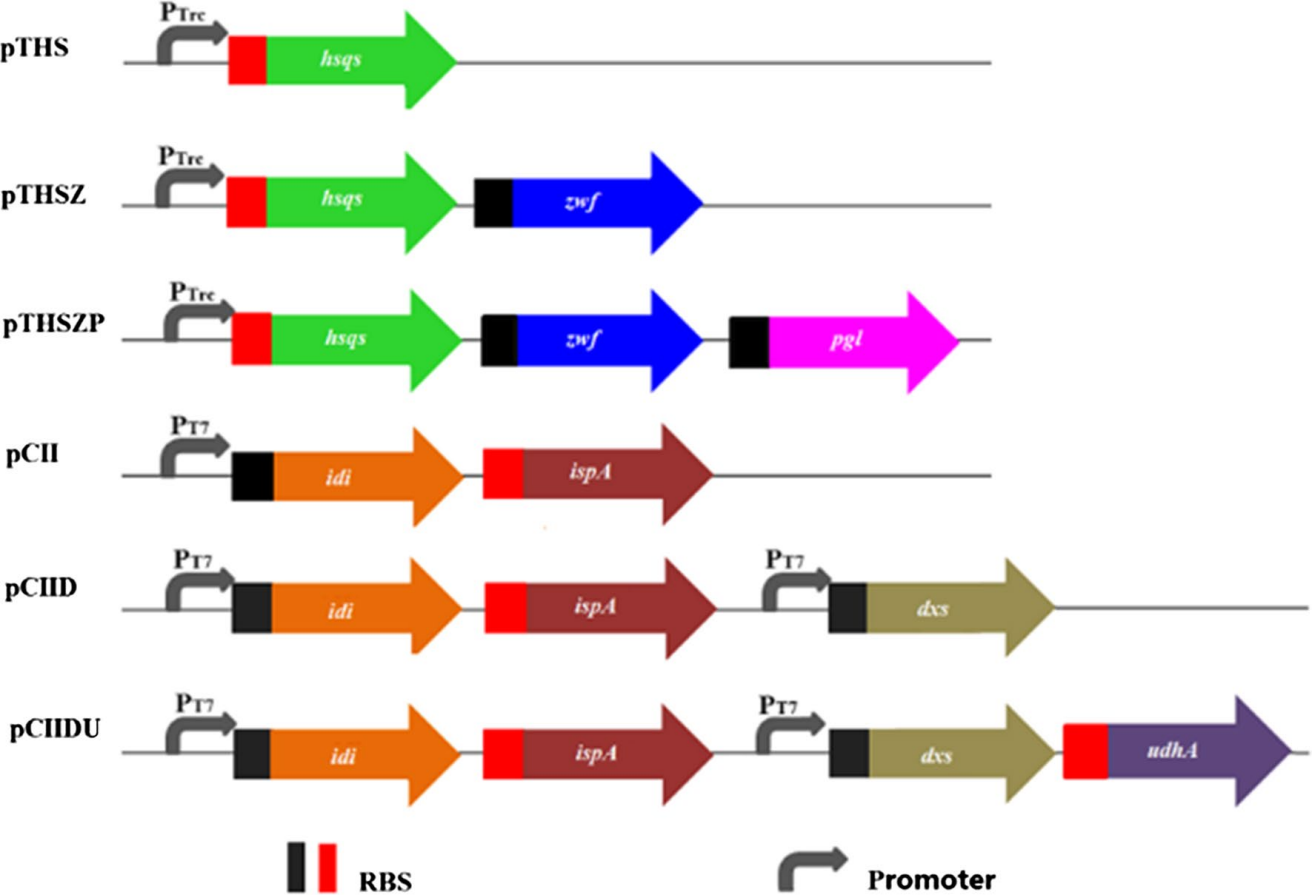
Structures of the constructed plasmids in this study. *RBS* ribosome binding site

General molecular manipulations were performed according to standard protocols [44]. For expression of *hsqs*, the *hsqs* gene was excised from pUC57-hsqs using *Nco*I and *Kpn*I, then ligated into plasmid pTrc99A cut with the same restriction enzymes to yield pTHS. For *zwf* expression, the sequence was PCR-amplified from the genomic DNA of *E. coli* using primer pair F_zwf_*Kpn*I/R_zwf_*Xba*I, and inserted into pTHS to yield pTHSZ. The *pgl* gene amplified using primer pair F_pgl_*Xba*I/R_pgl_*Hind*III, was inserted into pTHSZ between *Xba*I and *Hind*III sites, yielding pTHSZP. Genes involved in the isoprenoid biosynthesis such as *dxs*, *idi,* and *fps* were inserted into plasmid pCDFDuet-1. The *idi* and *fps* genes were PCR-amplified from *E. coli* genomic DNA using primer pair F_idi_*Nde*I/R_idi_OL and F_fps_OL/R_fps_*Kpn*I, respectively. Overlap extension PCR was used to assemble *idi* and *fps*, and the resulting PCR fragment was digested with *Nde*I/*Kpn*I, and inserted into plasmid pCDFDuet-1, to yield pCII. Subsequently, the *dxs* gene amplified using F_dxs_*Nco*I/R_dxs_*BamH*I, was inserted into pCII, yielding pCIID. The *udhA* gene encoding soluble pyridine nucleotide transhydrogenase, was excised from pUC57-*udhA* before insertion into pCIID between *Bam*HI and *EcoR*I restriction sites to yield pCIIDU. Notably, the 65th codon of the sequence was changed from GUU to GUG to prevent restriction by *EcoR*I. All constructed plasmids were sequenced to ensure their fidelity. The detailed information of plasmids and primers is listed in Additional file 1: Tables S2 and S3. Structures of plasmids are shown in Fig. 2.

### Deletion of chromosomal *pgi* and *menA* genes

The deletion of chromosomal *pgi* and *menA* genes was performed according to standard protocol [45]. For deleting the *pgi* and *menA* genes, two *kan*^*R*^ cassettes were amplified using the primers described previously [39, 46], with plasmid pKD13 as the PCR template. For homologous recombination, the linear *pgi* deletion cassette was introduced into *E. coli* JM109 (DE3)/pKD46, while the linear *menA* deletion cassette was introduced into *E. coli* JM109 (DE3) [*Δpgi*]/pKD46. After curing pKD46 at 42 °C, the help plasmid pCP20, encoding the FLP recombinase, was introduced into the recombinants to facilitate the removal of *kan*^*R*^ from chromosome. The candidate strains of *E. coli* JM109 (DE3) [*Δpgi*] and *E. coli* JM109 (DE3) [*Δpgi*, *ΔmenA*] were tested by sequencing.

### SDS-PAGE analysis of the protein profile

To verify the expression of hSQS in *E. coli*, SDS-PAGE was applied for analyzing the protein expression profile. To prepare protein samples, cell pellets collected by centrifugation (10,000*g*, 8 min) were resuspended in the lysis buffer containing phenylmethanesulfonyl fluoride (1 mM), Dnase (10 μg/mL) and lysozyme (0.2 mg/mL) and incubated at 37 °C for 30 min. Then, cell lysates were subjected to the ultrasonic treatment at 250 W for 20 min before they were resuspended in TE buffer and mixed with 5× Laemmli sample buffer. All the samples were analyzed by SDS-PAGE on a 15% polyacrylamide gel.

### Analysis of squalene, menaquinone, and demethylmenaquinone

Cell growth was measured using a spectrophotometer at 600 nm and converted to dry cell weight (DCW) upon a prepared standard curve of DCW versus OD_600_ (Additional file 1: Fig. S1). For squalene analysis, cells cultured for 32 h were harvested by centrifugation and freeze-dried. Total lipid extraction, including squalene from cells, was performed according to a previous method [47]. Squalene was analyzed by HPLC based on a previous method [48]. The procedure used to extract, separate, and analyze MK and DMK is described previously [49].

## Results and discussion

### Construction of a squalene-producing *E. coli* strain

**Fig. 3:**
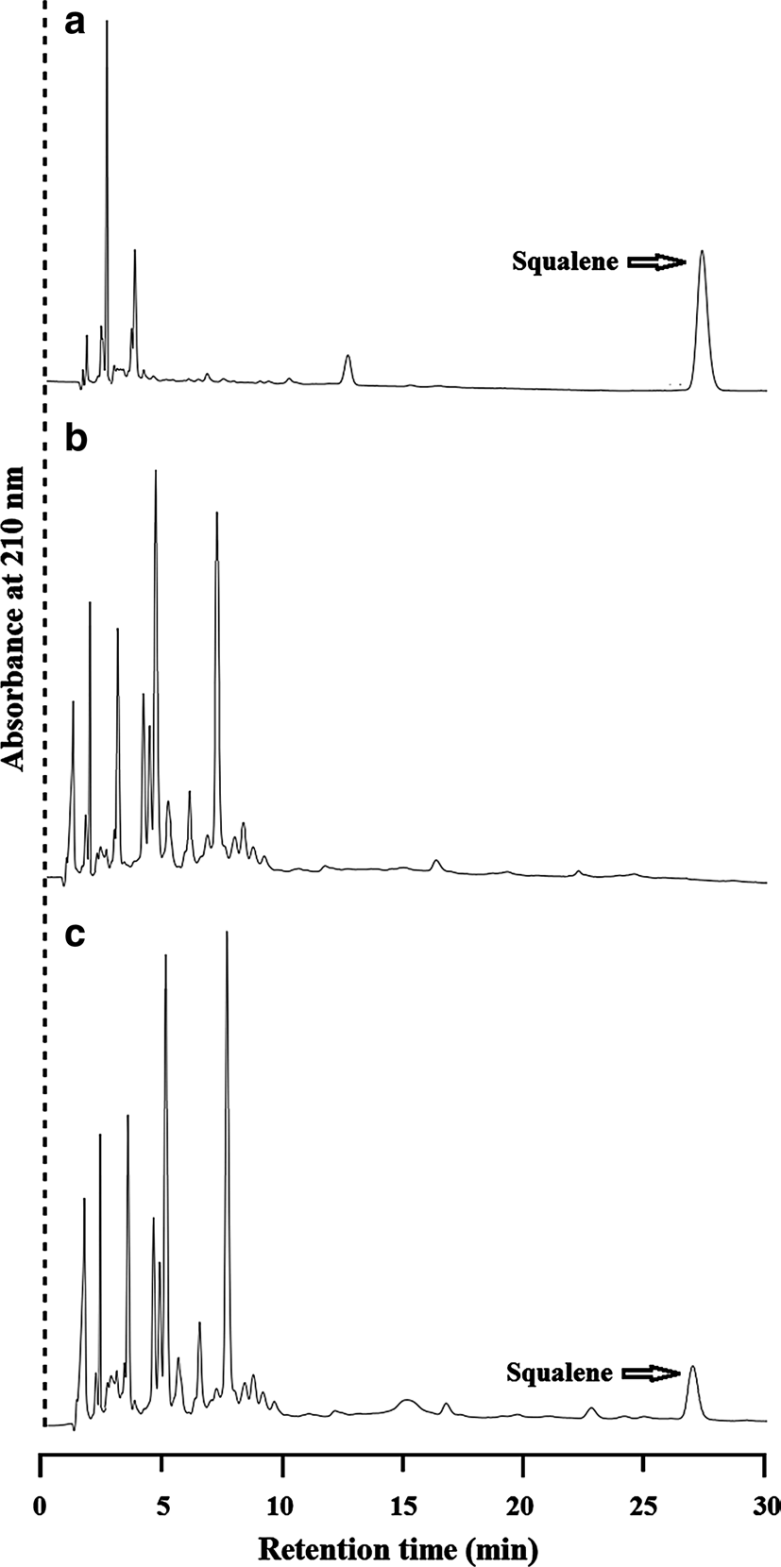
HPLC chromatograms of squalene production in different strains. **a** Squalene standard, **b** extract from ECHSQ0, **c** extract from ECHSQ1

In this study, the truncated human squalene synthase (hSQS) was employed for heterologous production of squalene in *E. coli* based on a previous report [19]. For an efficient *hsqs* expression, the cDNA sequence was optimized according to synonymous codon usage bias in *E. coli* (Additional file 1: Fig. S2). The sequence was then inserted into the plasmid pTrc99A to yield pTHS. Then, pTHS was introduced in *E. coli* JM109 (DE3) to construct ECHSQ1 strain. The expression of hSQS in ECHSQ1 was verified by SDS-PAGE, using ECHSQ0 harboring the empty vector pTrc99A as control (Additional file 1: Fig. S3). HPLC analysis confirmed that the *hsqs* expression in strain ECHSQ1 resulted in production of 1.37 mg/g dry cell weight (DCW) or 2.34 mg/L of squalene (Fig. 3), while it was undetectable in ECHSQ0 (Fig. 3). In the previous study [19], 4.2 mg/L of squalene was achieved by expressing *hsqs*. The difference in squalene production between our study and the previous study might be caused by using different host and expression vectors.

### Expression of rate-limiting enzymes in the isoprenoid pathway-enhanced squalene yield

In *E. coli*, isoprenoid biosynthesis begins with the formation of 1-deoxy-d-xylulose-5-phosphate (DXP). This is catalyzed by DXP synthase (DXS), a rate-limiting enzyme of the 2-C-methyl-d-erythritol 4-phosphate (MEP) pathway [50, 51] (Fig. 1). A series of other enzymes are used in subsequent reactions to convert MEP into the building blocks of isopentenyl diphosphate (IPP) and dimethylallyl diphosphate (DMAPP), which are further isomerized via isopentenyl diphosphate isomerase (IDI). Farnesyl diphosphate synthase (FPS) catalyzes the sequential 1′-4 coupling of IPP with DMAPP and geranyl diphosphate (GPP), resulting in the formation of FPP (Fig. 1). Based on the hypothesis that squalene synthase catalyzes fusion of two isoprenoid pathway farnesyl diphosphate (FPP) molecules into one squalene molecule over two consecutive steps, it was suggested that the FPP pool may be important for squalene synthesis [12, 20].

**Fig. 4:**
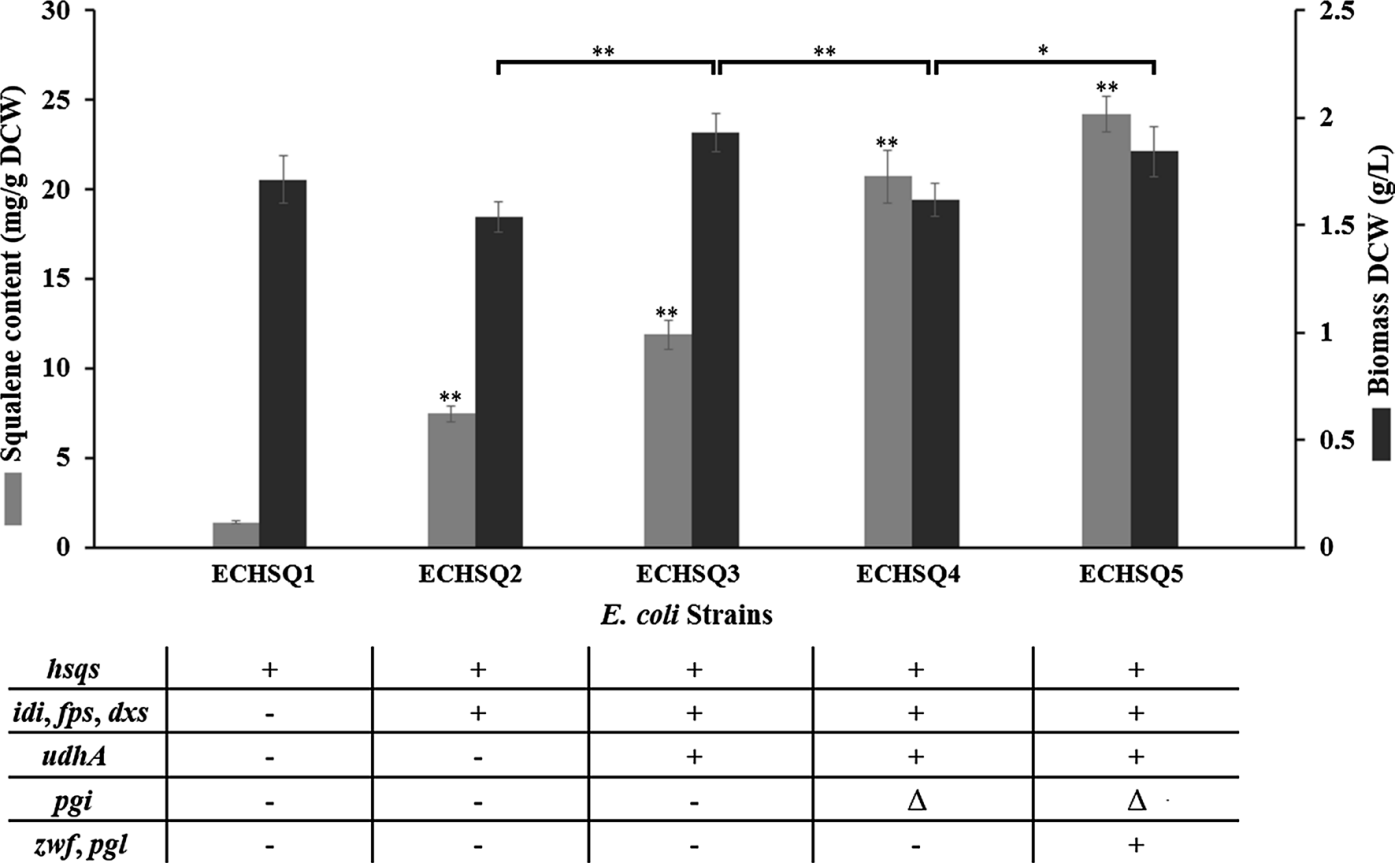
The total amount of squalene produced by different strains. *DCW* dry cell weight + overexpression, data represent the mean ± SD of duplicate samples in three separate experiments. Double asterisk significant difference (p < 0.01), Single asterisk significant difference (p < 0.05)

Upon successful construction of a squalene-producing *E. coli* strain, we investigated the effect of rate-limiting enzyme expression on increasing squalene content. Genes encoding rate-limiting enzymes including *dxs*, *idi,* and *fps*, which each have their own ribosome binding site (RBS), were inserted into pCDFDuet-1 under the control of a P_*t7*_ promotor to yield pCIID (Fig. 2). The recombinant strain ECHSQ2 harboring pCIID and pTHS showed a significant increase in squalene content (7.45 mg/g DCW), which was approximately fivefold increase over the ECHSQ1 strain (Fig. 4). These findings suggest that expression of rate-liming enzymes in isoprenoid pathway can benefit squalene synthesis by directing carbon flux towards the FPP.

### Increased squalene production resulted from a high NADPH/NADP^+^ ratio

Considering that enzymes involved in squalene synthesis, such as DXR, IspH and hSQS, require NADPH as a cofactor, we hypothesized the availability of NADPH/NADP^+^ might be important for squalene production. It has been reported that expressing specific enzymes could improve NADPH/NADP^+^ availability [25, 26, 31, 52]. One suggested method is to induce high expression of UdhA, which catalyzes reversible hydride transfer between NAD(H) and NADP(H), resulting in a significant increase in the NADPH/NADP^+^ ratio in *E. coli* [26]. To achieve high expression of UdhA, pCIIDU was constructed by inserting the *udhA* gene in pCIID, before it was introduced into ECHSQ2 to yield ECHSQ3. The squalene content of ECHSQ3 reached 11.86 mg/g DCW (22.8 mg/L), which was approximately 59% greater than strain ECHSQ2 (Fig. 4). Notably, the cell mass of ECHSQ3 was 25% greater than ECHSQ2, which was similar with the result from a previous study (Fig. 4) [35]. Our findings clearly indicate that the UdhA expression can increase squalene production in *E. coli* by supplying more NADPH/NADP^+^ for squalene biosynthetic enzymes. According to this study and previous research [26, 31–33, 53], a high NADPH/NADP^+^ ratio can affect the yields of various compounds.

### Increasing squalene production by modifying the isoprenoid pathway-feeding module and expressing rate-limiting enzymes

Based on the observation that EDP combined with PPP is a more efficient isoprenoid pathway-feeding module than the EMP [39], we hypothesized that squalene yield could be increased using EDP and PPP to feed the isoprenoid pathway. For blocking the EMP pathway and activating the EDP and PPP pathways, the *pgi* gene was deleted, generating the recombinant *E. coli* strain JM109 (DE3) [*Δpgi*]. To counteract the bottleneck of EDP and PPP, both *zwf* and *pgl* genes were expressed by assembling them into plasmid pTHS to yield pTHSZP.

The recombinant strain ECHSQ4 was constructed by introducing pCIIDU and pTHS into JM109 (DE3) [*Δpgi*]. HPLC analysis revealed that squalene production in ECHSQ4 was 20.7 mg/g DCW (33.5 mg/L), which was approximately 74% greater than strain ECHSQ3 (Fig. 4). Results suggest that EDP and PPP could be more beneficial for squalene synthesis than the EMP. This could be because G3P generation precedes pyruvate in EMP, causing an imbalance between the two precursors, which could decrease DXP production, consequently decreasing isoprene and squalene production. In EDP, G3P and pyruvate are generated simultaneously, causing an equal distribution between G3P and pyruvate pools to supply the isoprenoid pathway. To further enhance the effect of EDP and PPP on squalene production by counteracting the bottleneck, strain ECHSQ5 was constructed by introducing pCIIDU and pTHSZP into *E. coli* JM109 (DE3) [*Δpgi*]. Results show that squalene production was increased by a further 17% (24.2 mg/g DCW) due to the expression of *zwf* and *pgl* genes (Fig. 4). Note that the biomass of ECHSQ5 was increased from 1.62 to 1.83 g/L (Fig. 4), which suggests that glucose-6-phosphate dehydrogenase and 6-phosphogluconolactonase overexpression enhanced the cell’s ability to utilize glucose in the EDP and PPP for cell growth.

### Increasing squalene production by blocking the MK pathway

**Fig. 5:**
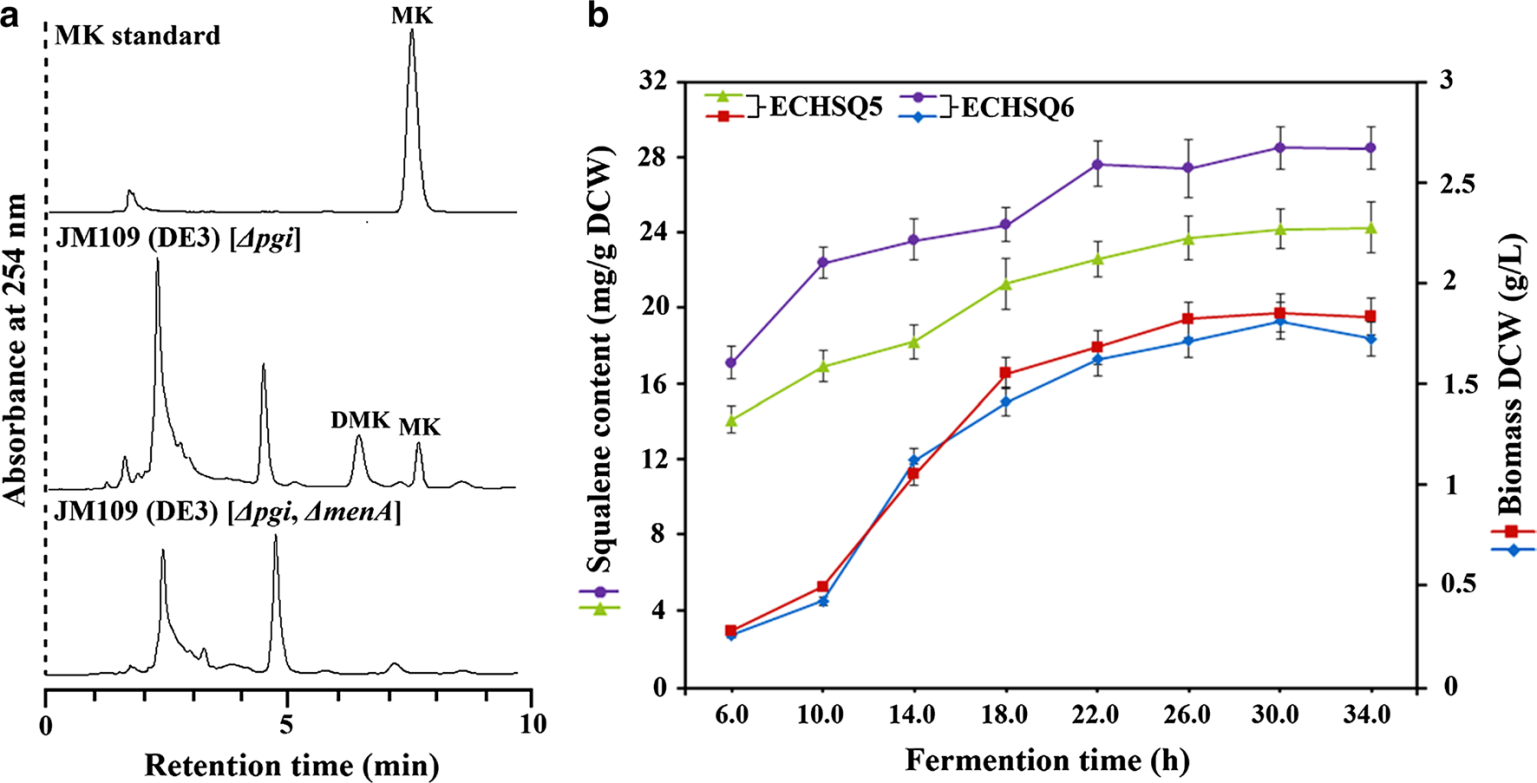
Effects of *menA* gene deletion on the biosynthesis of DMK, MK and squalene. **a** HPLC chromatogram of DMK and MK extracted from JM109 (DE3) [*Δpgi*] and JM109 (DE3) [*Δpgi*, *ΔmenA*]. **b** Squalene production in ECHSQ5 and ECHSQ6. Data represent the mean ± SD of duplicate samples in three separate experiments

DMK and MK have no essential function for aerobiosis of *E. coli* synthesis [43], but their synthesis occurs as a branched pathway of squalene synthesis, competing for the common precursor FPP (Fig. 1). Therefore, to supply more FPP for squalene biosynthesis, we blocked MK pathway by *menA* gene deletion to construct the recombinant strain JM109 (DE3) [*Δpgi*, *ΔmenA*]. The analysis results demonstrated the presence of DMK and MK in JM109 (DE3) [*Δpgi*]; however, both DMK and MK were not detected in JM109 (DE3) [*Δpgi*, *ΔmenA*] (Fig. 5a). This indicated that DMK and MK synthesis is blocked because of *menA* deletion. To further investigate the effect of MK pathway blocking on squalene accumulation, pCIIDU and pTHSZP were introduced into JM109 (DE3) [*∆pgi*, *∆menA*] to yield ECHSQ6 strain. The analysis results demonstrated that squalene content was increased from 24.2 mg/g DCW in ECHSQ5 to 28.5 mg/g DCW in ECHSQ6 in 32 h after fermentation (Fig. 5b). It is worth mentioning that the absence of MK and DMK had no negative effects on cell mass (Fig. 5b). And the production of squalene increased from 44.3 to 52.1 mg/L. The increase in squalene content suggested that FPP was directed toward the biosynthesis of squalene when the MK pathway was blocked. In this study, we demonstrate that the blocking of the branched pathway is an effective approach to improve the production of squalene.

## Conclusions

Squalene could be used instead of petroleum as a raw material for fuels [3, 4]. In the present study, we constructed a recombinant *E. coli* strain to produce squalene by expressing truncated human squalene synthase (hSQS), resulting in a squalene titer of 1.37 mg/g DCW. An approximately 21-fold improvement in squalene content (28.5 mg/g DCW) was observed via the overexpression of rate-limiting enzymes in isoprenoid pathway, the modification of isoprenoid-feeding module and the blocking of menaquinone pathway, providing efficient methods to increase the squalene yield in *E. coli*. This level of content is about half and tenfold of the reported values for *E. coil* [19, 54]. Besides, several other microorganisms have been employed as the producers of squalene. The value we obtained was about 1.5- and 1.8-fold of the highest values reported for engineered *S. cerevisiae* (15.8 mg/g DCW) [12] and *R. palustris* (18.5 mg/g DCW) [14], and was one-tenth of the natural squalene overproducer *Aurantiochytrium* sp. Yonez 5-1 (317 mg/g DCW) [18]. Although the yields of squalene achieved from *E. coli* in this study and previous studies [19, 54] were relatively lower compared to the natural squalene overproducer, *E. coli* is a competitive species for industrial applications because of its many advantages in production, such as short multiplication time, growth using inexpensive substrates, high-density fermentation and amenability to genetic modifications, etc. In the future, the combination of the strategies employed in this study and the methods developed in previous study [19] would create a synergistic effect on improving squalene yield in *E. coli*. Overall, this study provided novel strategies for improving squalene yield and demonstrated the potential of producing squalene by *E. coli*.

## Additional file

**Additional file 1.** Additional tables and figures.

## Abbreviations

DCW: Dry cell weight
mg/g: Mg per 1 g dry cell
mg/L: Mg per 1 L fermented liquid
HPLC: High-performance liquid chromatography
OD: Optical density
NADH: Nicotinamide adenine dinucleotide
NADPH: Nicotinamide adenine dinucleotide phosphate
RBS: Ribosome binding site
