# Supplementary material for: Improving squalene production by enhancing the NADPH/NADP+ ratio, modifying the isoprenoid-feeding module and blocking the menaquinone pathway in Escherichia coli
Source: Biotechnol Biofuels. 2019 Mar 28;12:68. doi: 10.1186/s13068-019-1415-x (PMC6437923; doi:10.1186/s13068-019-1415-x)
Supplement: Supplementary file 1 — Additional file 1. Additional tables and figures. [file 13068_2019_1415_MOESM1_ESM.docx]

**Additional Material**

**Improving squalene production by enhancing the NADPH/NADP^+^ ratio, modifying the isoprenoid feeding module and blocking the menaquinone pathway in *Escherichia coli***

Wen Xu^*1^ · Jia Yao^1^ · Lijun Liu^1^ · Xi Ma^1^ · Wei Li^1^ · Xiaojing Sun ^1^· Yang Wang^*1^

^1^The Molecular Virology and Viral Immunology Laboratory, Xi’an Medical University, Xi’an 710021, Shaanxi, China

*To whom correspondence may be addressed. Email: [yang.wang@xiyi.edu.cn](mailto:yang.wang@xiyi.edu.cn), [xuwen@xiyi.edu.cn](mailto:xuwen@xiyi.edu.cn)

**Table S1** List of strains used in the present study

| **Name** | **Genotype/description** | **Reference** |
| --- | --- | --- |
| **DH5α** | ***fhuA2Δ*(*arg*F-*lac*Z)U169 *phoA ginV44*** *Φ****80Δ*(lacZ)M15*gyrA96 recA1 relA1 endA1 thi-1 hsdR17*** | **Takara** |
| **JM109(DE3)** | ***endA1 recA1*gyr*A96 thi-1*hsd*R17 (rk-,mk+) relA1*sup*E44 D* (*lac-pro*AB) [F´*tra*D36*pro*AB*laqI*qZΔM15](DE3)** | Promega |
| **JM109(DE3)[*Δpgi*]** | chromosomal *pgi* gene deleted in **JM109(DE3)** | **This study** |
| **JM109(DE3)[*Δpgi, ΔmenA*]** | chromosomal *pgi* gene deleted in **JM109(DE3)[*Δpgi*]** | **This study** |
| ECHSQ0 | **JM109(DE3)**+pTrc99a | **This study** |
| ECHSQ1 | **JM109(DE3)**+pTHS | **This study** |
| ECHSQ2 | **JM109(DE3)**+ pTHS+pCIID | **This study** |
| ECHSQ3 | **JM109(DE3)**+ pTHS+pCIIDU | **This study** |
| ECHSQ4 | **JM109(DE3)[*Δpgi*]**+ pTHS+pCIIDU | **This study** |
| ECHSQ5 | **JM109(DE3)[*Δpgi*]**+ pTHSZP+pCIIDU | **This study** |
| ECHSQ6 | **JM109(DE3)[*Δpgi, ΔmenA*]**+ pTHSZP+pCIIDU | **This study** |

**Table S2** List of plasmids used in the present study

| Name | Description |
| --- | --- |
| pUC57 | Cloning vector |
| pCDFDuet-1 | Low copy expression vector; pCDF origin; Sp^R^ |
| pTrc99a | Low copy expression vector; pBR322 origin; **Ap^R^** |
| pUC57-hsqs | pUC57 derivative with codon-optimized *hsqs* gene synthesized by GeneScript |
| pTHS | pTrc99a derivative with the *hSapB* gene under the control of Trc promoter |
| pTHSZ | pTHS derivative with the *zwf* gene under the control of Trc promoter |
| pTHSZP | pTHSZ derivative with the *pgl* gene under the control of Trc promoter |
| pCII | pCDFDuet-1 derivative with the genes of *idi* and *ispA* under the control of T7 promoter |
| pCIID | pCII derivative with the *dxs* gene under the control of T7 promoter |
| pCIIDU | pCIID derivative with the *udhA* gene under the control of T7 promoter |

**Table S3** List of oligonucleotides used in the present study

| Name | Discription |
| --- | --- |
| F_zwf_*Kpn*I | GGGGTACCAAGGAGATATAATGGCGGTAACGCAAACAGCC |
| R_zwf_*Xba*I | GCTCTAGATTACTCAAACTCATTCCAGGA |
| F_pgl_*Xba*I | GCTCTAGAAAGGAGATATAATGAAGCAAACAGTTTATATCG |
| R_pgl_*Hind*III | CCCAAGCTTTTAGTGTGCGTTAACCACCAC |
| F_check_99A | ATGGGCGGTAAACTGCTGCAG |
| R_check_99A | AAGCTTGCATGCCTGCAGGTC |
| F_idi_*Nde*I | GGAATTCCATATGCAAACGGAACACGTCATTTTATTG |
| R_idi_OL | ATGTATATCTCCTTTTATTTAAGCTGGGTAAATGCAGATAA |
| F_fps_OL | TTATCTGCATTTACCCAGCTTAAATAAAAGGAGATATACATATGGACTTTCCGCAGCAACTCGAAGC |
| R_fps_*Kpn*I | GGGGTACCTTATTTATTACGCTGGATGATGTACG |
| F_check_CD1 | GGATCTCGACGCTCTCCCT |
| R_check_CD1 | GATTATGCGGCCGTGTACAA |
| F_dxs_*Nco*I | CATGCCATGGGCATGAGTTTTGATATTGCCAAA |
| R_dxs_*BamH*I | CGGGATCCTTATGCCAGCCAGGCCTTGATT |
| F_check_CD2 | AATTAATACGACTCACTATAG |
| R_check_CD2 | ATGCTAGTTATTGCTCAGCGGTGGCA |

**
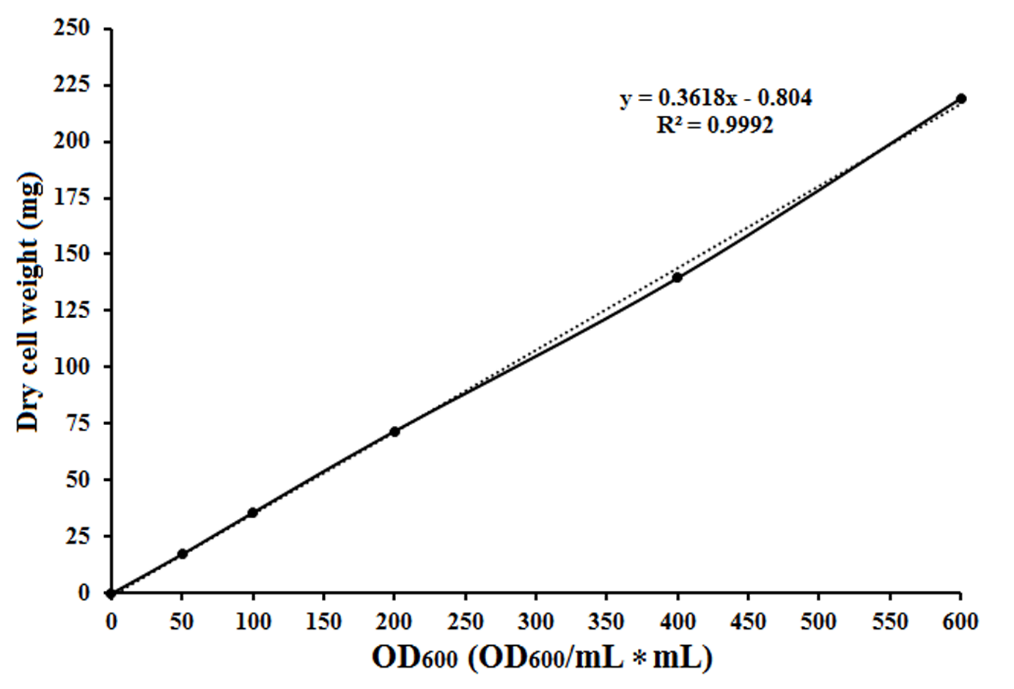
**

**Figure S1** The standard curve of DCW versus OD600.

**
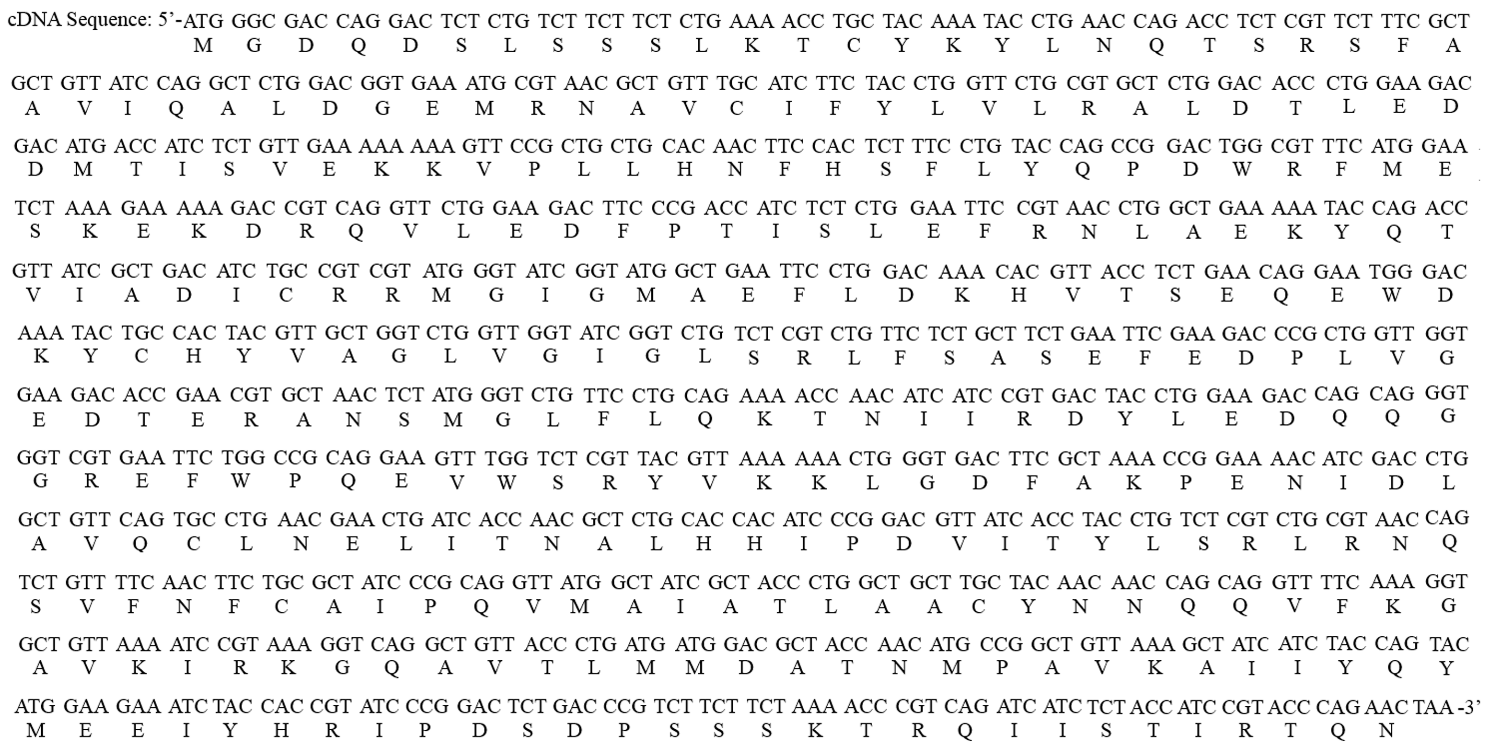
**

**Figure S2** Codon optimized gene of human *hsqs* for overexpression in ***Escherichia coli***.


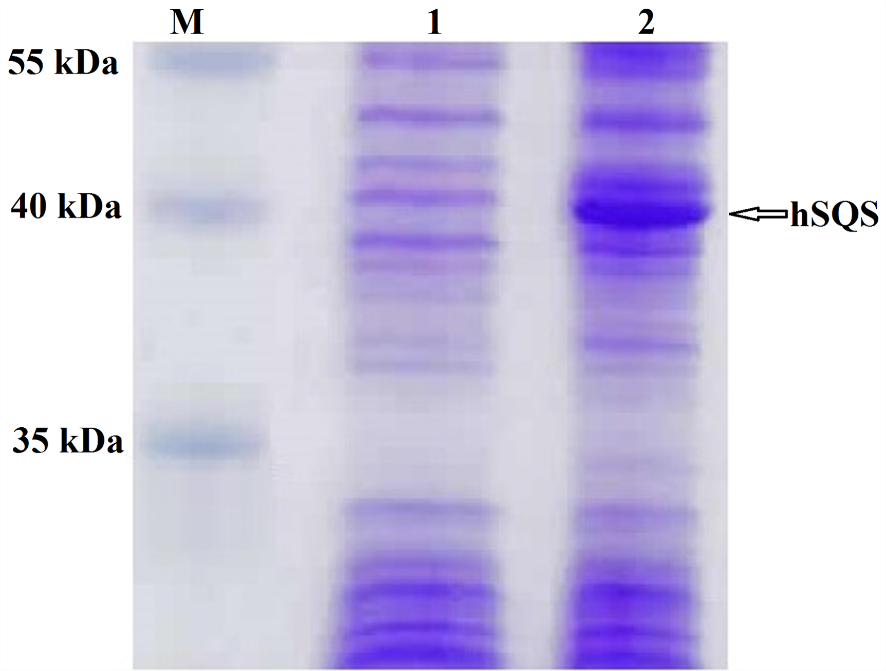


**Figure S3** SDS-PAGE analysis of hSapB overexpression. *M* PageRuler Prestained Protein Ladder, *1* proteins from the whole cell of ECHSQ0, *2* proteins from the whole cell of ECHSQ1.
